# Motor Skill Improvement Using Compressive Garments in a Child with Multidimensional Impairments and Ehlers–Danlos Syndrome

**DOI:** 10.1155/2022/5819406

**Published:** 2022-09-06

**Authors:** Jean Xavier, Soizic Gauthier, Ingrid Zammouri, Salvatore Maria Anzalone, David Cohen

**Affiliations:** ^1^Department of Child and Adolescent Psychiatry, Henri Laborit Hospital Centre, 86000 Poitiers, France; ^2^CNRS UMR 7295, Équipe CoCliCo, Cognition and Learning Research Center, Poitiers, France; ^3^Department of Child and Adolescent Psychiatry, Reference Centre for Rare Psychiatric Diseases, AP-HP, Groupe Hospitalier Pitié-Salpêtrière, Sorbonne Université, 75006 Paris, France; ^4^Laboratoire CHArt-THIM, EA4004, Université Paris 8, Saint-Denis, France; ^5^CNRS UMR 7222, Institute for Intelligent Systems and Robotics, Sorbonne Université, 75006 Paris, France

## Abstract

We report the case of an 8-year-old child with a complex neurodevelopmental disorder, including severe developmental coordination disorder with dysgraphia, anxiety and depression, mild social functioning impairments, headache and chronic musculoskeletal pain, secondary to Ehlers–Danlos syndrome (EDS) hypermobility type. We explored whether wearing whole-body compressive garments (CGs) could improve his motor skills assessed through standardized and experimental procedures. In addition to the effectiveness of CGs on pain, we found partial improvements in his motor skills, specifically postural control, hand movements, and body schema representation, after wearing CGs for 15 days. During an experimental motor imitation task with a virtual tightrope walker, we found improvements in interpersonal synchronization with performances closer to those of typical developing (TD) controls. We conclude that CGs appear to be an innovative and interesting adjuvant treatment for motor skill impairments in children with multidimensional impairments involving EDS. These promising results require confirmation by further evidence-based research.

## 1. Introduction

Neurodevelopmental disorders (NDDs) are a group of conditions (e.g., attention-deficit/hyperactivity disorder (ADHD), autism spectrum disorder (ASD), developmental coordination disorder, tic disorder, and learning disorder) with onset in infancy or childhood. The disorders are characterized by developmental deficits that produce impairments of personal, social, academic, or occupational functioning (DSM-5, [1]). NDDs may include the specifier “associated with a known medical or genetic condition or environmental factor,” offering the clinician the possibility to document etiological factors. Heritable disorders of connective tissue, characterized by tissue fragility and joint hypermobility, include Ehlers–Danlos syndrome (EDS). The hypermobility type of EDS (EDS-HT) constitutes the most common disorder of connective tissue leading to systemic damage. It is characterized by tissue fragility, joint hypermobility, and multisystem problems that usually appear in childhood [2]. A strong association exists between EDS-HT and several coexisting developmental difficulties, notably in the field of NDDs [3]. These difficulties relate to different domains, such as communication, language, social interrelatedness, motor coordination and behavior, and fall within the Early Symptomatic Syndromes Eliciting Neurodevelopmental Clinical Examinations (ESSENCE) framework created by Gillberg [4]. ESSENCE was proposed to stress the need to explore clusters of neurodevelopmental problems often screened separately in discrete diagnoses (e.g., autism spectrum disorder (ASD) or developmental coordination disorder (DCD)), although they are highly coexisting. For example, both of these disorders include social interaction difficulties (at the core of ASD) associated with motor impairments (at the core of DCD). ESSENCE problems are present in a significant number of children with EDS-HT: motor and balance problems, hypotonia, and proprioceptive impairments [2]. Whole-body compressive garments (CGs) have been specifically designed for patients with EDS and are currently used to reduce proprioceptive dysfunction associated with EDS, to relieve pain and fatigue, and to improve mobility [5]. In individuals with ASD and challenging behavior comorbid with severe proprioceptive dysfunction associated or not with EDS, CGs appeared to be of interest in reducing these comorbid challenging behaviors and improving motor coordination [6]. Here, we report the case of an 8-year-old child with NDD symptoms and EDS. We used the framework of ESSENCE to address him as a child with multidimensional impairments [7]. Given the severe proprioceptive dysfunction, we explored whether wearing CGs could improve his motor skills using both standardized and experimental procedures.

## 2. Methods

### 2.1. Participant

This is a single-case design study. P. is a single 8-year-old boy of unrelated parents. His father had essential tremor, and his mother had breast cancer in remission declared when P. was 6. He also had two brief psychotic disorders at 17 and 20 years old. P. was born by vaginal delivery at 41 weeks of amenorrhea. Pregnancy was uncomplicated. The mother was 31, and the father was 29 at conception. His birth weight was 3010 g, length was 50 cm, and head circumference was 34 cm with plagiocephaly. P. was hospitalized the first 10 days of life for a maternal fetal infection with *Streptococcus B*. His history was significant with regard to motor development. He walked at 16 months and had difficulty with fine motor skills and motor coordination associated with hypotonia and speech delay (first words at 1 year; first sentences at 3 with pronunciation difficulties). At age 3, he had a tendency to avoid eye contact, and keeping up a conversation with him was difficult. Furthermore, he developed difficulty falling asleep and experienced repeated awakenings. Given his speech impairments and motor difficulties, P. began speech and occupational therapy at age four. In addition, P. had presented social interaction difficulties from the nursery where he was described as being isolated from the other children. When he started learning to read and write, he showed writing sluggishness and motor tremor.

In terms of medical history, P. had asthma, gastroesophageal reflux that prevented breastfeeding, repeated ear infections and conjunctivitis. From 18 months to five years of age, P. had seven episodes of generalized hyperthermic tonic–clonic seizures. Electroencephalography (EEG) while awake at four years of age was normal. A cerebral scan found an infracentimetric cyst in the frontal horn wall with a regular, homogeneous, noncompressive, banal appearance. From the age of five, P. presented episodes of facial urticaria during cold and wet periods without fever that was treated with antihistamines. These episodes were inconsistently associated with 24 h periods of gonalgia. At seven, his physical examination revealed statural acceleration (at +2 standard deviations), asymmetry of the lower limbs and scoliosis attitude and skin hyperlaxity and hyperelasticity. A cryoglobulin test detected polyclonal type II cryoglobulinemia.

When we met P., he was 8 years old and referred to our outpatient unit for several reasons: social interaction difficulties associated with motor disabilities and a high number of somatic symptoms during his development. He had glasses for nearsightedness and astigmatism. He had oculomotor therapy and psychotherapy. To organize a care project addressing the complexity of his pathology, we used both standardized and experimental procedures.

### 2.2. Standardized Assessments

To assess P., we used the following standardized procedures. To score autism core symptoms, we used the Autism Diagnostic Interview-revised (ADI-R) [8] and the Autism Diagnostic Observation Schedule (ADOS) [9]. For general cognition, we used the Wechsler Intelligence Scale for Children- (WISC-) V [10]. For the motor dimension that was the most complex based on clinical examination, we used several assessments: the Movement Assessment Battery for Children (MABC-2) [11], evaluation of distal gnosopraxis (EMG) [12], the Frostig developmental test of visual perception [13], spatial orientation [14], and the Goodenough draw-a-man test [15]. In addition, the EDS diagnosis was based on the criteria reported by Malfait et al. [16]. We used the Beighton score to identify joint hypermobility.

### 2.3. Experimental Procedure

**Figure 1:**
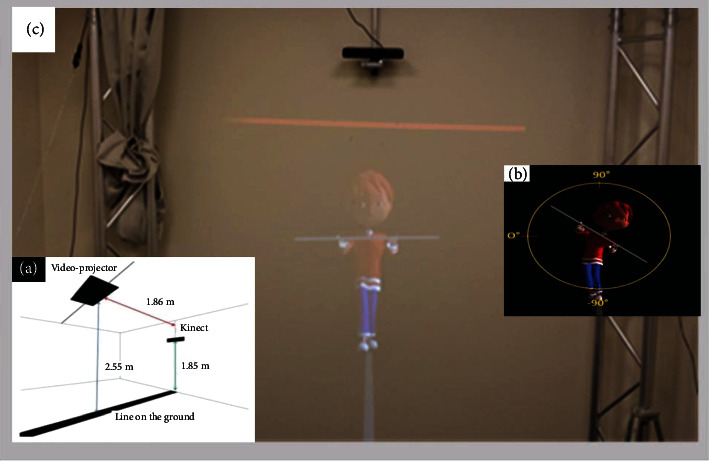
Principles and setup of the experiment. (a) Schematic illustration of the experimental room with the projection on the wall of the tightrope walker avatar. (b) Tightrope walker avatar's head and bar inclinations in the front-facing orientation.

The tightrope walker (TW) paradigm [17] is an experimental setup designed to test the ability to change spatial viewpoints during a spontaneous motor imitation task. We adapted the paradigm to children by adjusting the size of the TW and giving the TW a cartoon-like aspect of a child. We developed a 3D animation where a 3D character is walking on a rope and holding a bar in front of him (Figure 1). The animated TW was displayed life-sized by a rear projector onto a large screen (2∗2 m). The TW was 0.81-meter high when standing in the middle part of the rope and 1.13-meter high when he was “closest” to the participant. To mimic everyday social encounters and reinforce interactions that give the participants the impression of acting in the same spatial environment as the TW, the participants stood on a black line on the ground that was continuous with the avatar's rope on the screen (Figure 1(a)). Before the movie started, we asked participants to find a comfortable position, with their legs slightly apart, and to not shift from their position in response to the moves of the TW. The participants held a wooden bar horizontally in front of them. During the experimental procedure, the TW is first shown in a front-facing orientation, standing with his right foot in front of the left on the rope for the first 30 s. Then, during the subsequent 7 trials, numbered from 1 to 7, the TW alternately walked either towards or away from the participant in two orientations: (i) a front-facing orientation when the TW walked forward and (ii) a back-facing orientation when the TW was walking away, and the participants saw the TW from his back. While walking in each orientation, the TW executed lateral tilts with his bar either to his right or his left in random order (Figure 1(b)). The participants were instructed to observe the tilts of the TW and to lean when he is leaning. For details of the experimental procedure, see the experimental procedure in Xavier et al. [18].

## 3. Results

### 3.1. Clinical Assessment

P. had difficulties in starting and maintaining relationships with peers. He experienced school bullying that affected his mood but had good academic performance despite difficulties writing. He reported constant anxiety and expressed the fear that people would break into his home to harm him. In addition, he did not like the unexpected and needed to know the schedule of his days in advance. Based on parental reports, P. was often “in his world” with a special interest in metro stations and lines. P. also had intense periumbilical abdominal pain with nausea, dizziness, or phonophobia sometimes associated with headaches. He reported cramp-like knee pains and a significant amount of exercise fatigue. We confirmed EDS and obtained a Beighton score for joint hypermobility equal to 6.

**Figure 2:**
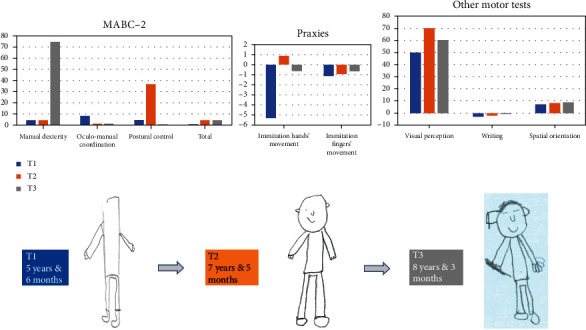
Evolution of motor skills in the three times of standardized assessment (MABC-2 is given in percentile. Praxies from EMG are given in standard deviation).

Table 1 reports assessments related to autism and cognition. The autism assessment scores did not support a diagnosis of ASD. Regarding the WISC-V, the most striking feature was the discrepancy between indexes: the verbal comprehension index was in the superior range, and the other indexes were in the average range with the exception of processing speed, which was in the low average range. The score obtained for coding was just below the average and was partially explained by P.'s writing difficulties. The full-scale IQ computation was invalid because of the degree of heterogeneity. Standardized motor assessments were confirmed DCD (see below and Figure 2). Therefore, the psychiatric diagnosis was complex and included several neurodevelopmental conditions (phonological disorder and severe DCD with dysgraphia), social anxiety disorder, and episodes of depression associated with EDS and bullying.

### 3.2. Motor Skills and Improvements with Whole-Body CGs

CGs are currently funded by the French healthcare system with an indication for EDS. P's CGs were customized based on his need by an orthotic and prosthetic practitioner from Novatex Medical. The CGs included tailor-made pants, a vest, and mittens, which covered his entire body. As expected in EDS, P. reported improvements in knee pain by wearing the CGs. Figure 2 shows three times of standardized assessments including T1 (without CGs), a partial improvement in motor skills on T2–15 days after starting to wear CGs (at least 2 h/day), and T3–15 days after stopping wearing CGs.

MABC-2 total scores remained pathological (<5^th^ percentile) at all time points. However, postural control significantly improved at T2, with a score moving from the 5^th^ to the 37^th^ percentile, although this score returned to pathological levels at T3, whereas manual dexterity improved only at T3, moving from the 5^th^ (at T1 and T2) to the 75^th^ percentile. Distal gnosopraxis (EMG) remained subnormal for finger movements (starting from -1.13 SD at T1 to -0.91 SD at T2 and -0.67 SD at T3). However, scores dramatically improved for hand movements starting from -5.29 SD at T1 to +0.09 SD at T2 and -0.67 SD at T3. P.'s developmental level regarding a representation of body schema through the draw-a-man test also dramatically improved after wearing CGs. He had a developmental age of 5 years and 6 months at T1, which was a gap of nearly 2 years with respect to his chronological age. After wearing the CGs, P.'s developmental age reached 7 years and 5 months at T2 and 8 years and 3 months at T3. Finally, writing and spatial orientation performance did not change and remained pathological. Visual perception was in the normal range and remained as such.

**Figure 3:**
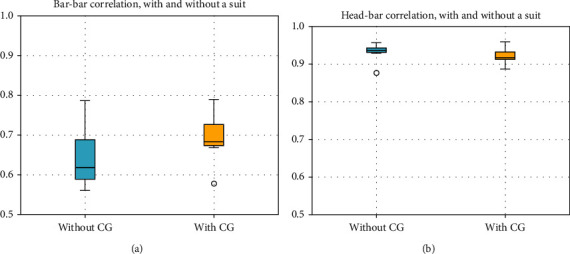
P's bar x TW bar correlation (3a) and P's head x P's bar correlation during the tightrope walker (TW) experiment.

**Figure 4:**
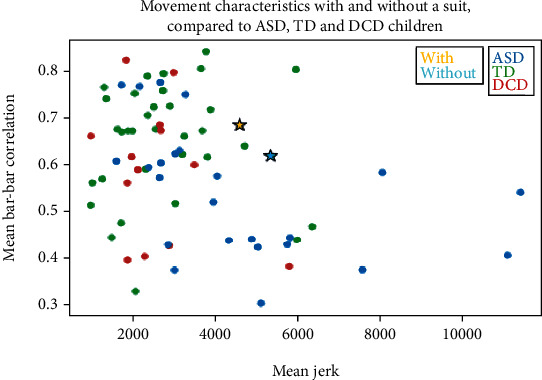
P's position according to mean bar x bar correlation (*y*) and mean jerk (*x*) during the tightrope walker experiment compared to TD controls (green dots) and children with ASD (blue dots) or DCD (red dots). TD: typical developing; ASD: autism spectrum disorder; DCD: developmental coordination disorder.

Figure 3(a) shows the interpersonal synchronization between P. and TW during the experimental procedure as measured by the correlation coefficient between P.'s bar angle and the TW's bar angle. The correlation significantly increased after wearing CGs (T2) compared to T1.  Figure 3(b) shows P.'s motor coordination as measured by the correlation coefficient between P.'s bar angle and his head axis angles during the experiment. As expected, given the tendency to move one's head in the same direction of the bar during this type of experimental procedure, the correlation was excellent (>0.9) and did not change after wearing CGs. Finally, we compared P.'s results with and without wearing CG to 70 participants aged 6 to 18 years, including typical developing (TD) controls and children with ASD or DCD who participated in the same experimental procedure [18, 19].  Figure 4 illustrates the mean bar-bar correlation and mean jerk during the experiment. It appears that with the CGs, P.'s position in space was slightly more restricted than the space in which most TD controls were (upper left side of the square).

## 4. Discussion

To the best of our knowledge, this is the first clinical study assessing the impact of whole-body CGs on the motor skills of a child from a semiecological point of view through a spontaneous motor imitation task and a psychomotor evaluation. P. is an 8-year-old child with a complex developmental disorder associated with an EDS hypermobility type. In the context of this multisystem disease [20], he had several somatic symptoms (hyperextensible skin, scoliosis, myopia, and gastrooesophageal reflux) associated with headache, chronic pain of musculoskeletal origin (arthralgia and myalgias), and fatigue. These symptoms associated with postural deficiency syndrome and DCD contributed to P.'s difficulties in terms of negative emotions, social functioning, and dysgraphia [21–23]. According to the ESSENCE framework, EDS is associated with coexisting emotional problems and NDDs such as DCD.

Beyond the effectiveness of whole-body CGs on his knee pain, the CGs also partially improved P's motor skills. Regarding the comorbidity between DCD and EDS, P. had deficits in proprioception [24], which could account for difficulties in postural control and balance [25] and be involved in motor control abnormalities [26]. Postural control is integral to the execution of an action, ensuring the maintenance of equilibrium during movement. Posture is a reference frame for the production of accurate movements [27]. Based on the three standardized assessments, as expected, postural control was improved only when P. was wearing CGs. More surprising was the dramatic improvements in (1) hand movements scores and (2) developmental levels regarding body schema representation after wearing CG for 15 days. Improvement (1) is consistent with the relationship that exists during child development between postural control (which offer head and trunk stability) and eye/hand coordination [28]; improvement (2) is explained by the fact that proprioceptive and kinesthetic information participates in body awareness and body schema representation [29]. During T3, despite not wearing the CGs in the previous 15 days, the improvement in manual dexterity and the remaining improvement in T2 scores concerning hand movements could be understood as the result of a retest effect. During T2, wearing his whole-body CGs, P. experienced new motor abilities and cognitively integrated the test contents he performed. We can also postulate that this integration was made through proprioceptive memory allowing him to answer again at T3, in a manner similar to T2 where he performed the same tasks. As a result of his DCD and to the extent that the writing test is a timed test, scores on this test remained pathological.

We found improvements in interpersonal synchronization (correlation between P.'s bar angles and the TW's bar angles) with CGs, which was allowed by the improvements in P.'s postural control mentioned above.

Finally, P.'s results, with and without wearing the CGs, were compared to those of 70 participants divided into 3 groups (TD controls and children with ASD or DCD) who participated in the same experimental procedure [18]. The authors found that the ASD group had lower performance in interpersonal synchrony (bar-bar correlation) than both the TD and DCD groups. ASD individuals also had lower motor control performance. These results may be related to impairments described in this population in terms of (1) motor control, which manifests by more jerky and less accurate movements [30] and (2) postural control with meaningful variability in posture [31]. With CGs providing improvements in his postural control, we found that P.'s performances, in terms of interpersonal synchrony and jerk, came closer to those of TD controls.

## 5. Conclusion

As illustrated by this case report, a multidimensional perspective allows one to address the child holistically, taking into account his coexisting developmental difficulties. This approach allows clinicians to achieve a functional diagnosis enabling the elaboration of a tailored therapeutic plan and better school inclusion. CGs appear to be an innovative and interesting adjuvant treatment for motor skill impairments in children with multidimensional impairments involving ESD. These promising results require confirmation by further evidence-based research, not only focusing on motor skills but also taking into account other developmental aspects, such as emotion regulation and social interaction abilities.

## Figures and Tables

**Table 1:** Summary of interdisciplinary assessments in 8 years old boy with an atypical autism and an Ehlers–Danlos Syndrome.

General cognition–WISC-V	Scores	*Comments*
Verbal comprehension index	118	Heterogeneous profile does not allow calculation of total IQ
Similarities	13	
Vocabulary	14	
Visual spatial index	100	
Block design	9	
Visual puzzles	11	
Fluid reasoning index	103	
Matrix reasoning	13	
Figure weights	8	
Working memory index	97	
Digit span	9	
Picture span	10	
Processing speed index	86	
Coding	7	
Symbol search	8	
Autism diagnostic interview (ADI-R) ^∗^		
Social domain (cutoff score ≤ 10)	6	All symptoms were mild. Communication domain, stereotyped behavior domain and the developmental domain are above the ADI-R cutoff. Social domain score is under the cutoff score.
Communication domain and language (cutoff score ≤ 8)	8	
Stereotyped behavior domain (cutoff score = 3)	3	
Developmental domain 5 (cutoff score = 1)	2	
Autism diagnostic observation schedule ADOS (module 3)	6	Scores obtained are all under the cutoff for ASD
(cutoff score for ASD = 7)		
Communication	1	
Reciprocal social interaction	4	
Restricted and repetitive behavior domain	1	

WISC–V: Wechsler Intelligence Scale for Children–V; ADI-R: Autism Diagnostic Interview–Revised; ASD: autism spectrum disorder; IQ: intellectual quotient. ^∗^ADI-R scores were assigned according to clinical impairments at age 5 years.
